# Preparation of ZnO@TiO_2_ nanotubes heterostructured film by thermal decomposition and their photocatalytic performances†

**DOI:** 10.1039/c7ra13222k

**Published:** 2018-02-20

**Authors:** Yulong Liao, Kaibin Zhang, Xiaoyi Wang, Dainan Zhang, Yuanxun Li, Hua Su, Huaiwu Zhang, Zhiyong Zhong

**Affiliations:** State Key Laboratory of Electronic Thin Film and Integrated Devices, University of Electronic Science and Technology of China Chengdu 610054 China yulong.liao@uestc.edu.cn +86-028-83202556 +86-028-83201440; Center for Applied Chemistry, University of Electronic Science and Technology of China Chengdu 610054 China

## Abstract

TiO_2_ nanotubes (NTs) arrays prepared by anodic oxidation were modified with ZnO particles and their morphology and photocatalytic properties were investigated. A simple thermal decomposition process was involved in the modification method. Zinc acetate solution was filled into the TiO_2_ NTs arrays, and ZnO@TiO_2_ heterojunction films were formed after the thermal treatment. The morphology and catalytic properties of the heterojunction films could be manipulated by the concentration of zinc acetate solution. Compared to TiO_2_ NTs arrays, the ZnO@TiO_2_ heterojunction films with an optimized concentration of zinc acetate showed enhanced catalytic performances. Their photocatalytic activities were discussed with respect to the formation of ZnO@TiO_2_ heterojunctions and enforced charge separation. This study demonstrates a simple method to prepare ZnO nanoparticles@TiO_2_ NT heterojunction films, which is promising for other environmental and energy related applications.

## Abstract 2

Efficient and low-cost preparation method of ZnO nanoparticles@TiO_2_ NT heterojunction films, has a positive effect on water pollution control.

## Graphical abstract

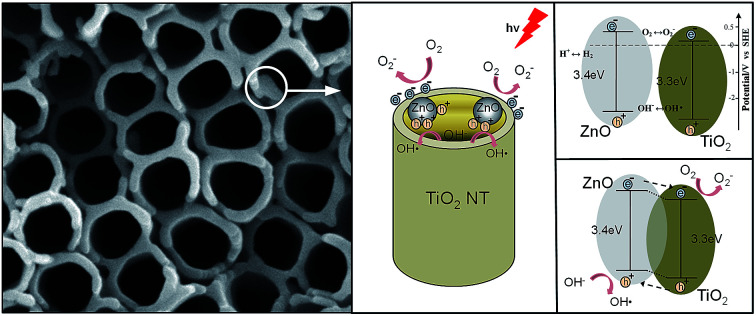

## Introduction

In recent years, the environment has been deteriorated due to pollution, while energy innovation is imminent. With such a trend, nanostructured semiconductors have scope for development and are in demand.^1,2^ Nanoscale metal oxides can be applied in numerous areas, such as in clean and recyclable energy.^3–6^ Among the nano-metal oxides, ZnO and TiO_2_ were widely adopted as photoelectric material and applied in the photovoltaic (PV) and photoelectrochemical (PEC) methods. TiO_2_ can not only be used as a catalyst for the treatment of water pollution, but also as a new type of solar cell materials.^7,8^ Ordered TiO_2_ array can produce higher charge collection efficiency owing to a direct conduction path for electrons when used as photo electrodes.^9–11^ In order to avoid recycling problems, TiO_2_ nanomaterials in powder form were gradually being eliminated, while TiO_2_ NTs have attracted increasing attention. TiO_2_ NTs arrays have several advantages as photocatalysts, such as simple synthetic craft, cheap cost and high stability.^12,13^ Nanotubes have a greater specific surface area than bulk materials and therefore, have a higher adsorption capacity. In particular, if these NTs can be filled with inorganic, organic, metal or magnetic nanoparticles in nano-scale, it will greatly improve their photoelectric, electromagnetic, and catalytic performances. In the field of pollution monitoring, metal oxides such as TiO_2_ and ZnO have received the attention of researchers due to their attractive photocatalytic performance. Pure TiO_2_ NTs have limited photocatalytic activity without visible light response, as reported in former studies,^14,15^ owing to their wide band gap (*E*_g_ ∼ 3.3 eV) and high recombination rate of photogenerated electron–hole pairs.^16–20^ TiO_2_ NTs need to be decorated or modified to achieve better performance. ZnO stands out as a modifier material. ZnO is one of the most promising and demanding functional materials for its good flexibility in synthesis and morphology.^21–23^

For photocatalytic reaction, the conduction band edge of ZnO is more negative than TiO_2_, which makes ZnO an ideal material for enhancing the photoelectrochemical performance by coupling with TiO_2_.^24–26^ ZnO and TiO_2_ have similar bandgap energies (TiO_2_ ∼ 3.3 eV, ZnO ∼ 3.4 eV). Their level structures are mutually satisfied for constitution of heterogeneous structural materials.^27–29^ Heterogeneous ZnO@TiO_2_ nano-composites have exhibited excellent performances partly due to the high reactivity of TiO_2_ and the high electron mobility of ZnO, which improve the process of electrons and holes transfer between the corresponding conduction and valence bands.^30–34^ Compared to pure TiO_2_ NTs arrays, heterogeneous ZnO@TiO_2_ NTs arrays are expected to have a greater light-response and higher collection efficiency.

Thus far, ZnO@TiO_2_ heterojunctions have been successfully prepared by hydrothermal process,^35^ spin coating,^36^ electrospinning and sol–gel processes.^37^ In this study, a new synthesis approach is proposed: TiO_2_ NT micro-containers template thermal decomposition method. TiO_2_ NTs arrays were prepared by anodic oxidation method and filled with various concentrations of zinc acetate solution. This method is found to be extremely simple and efficient. The TiO_2_ nanotubes simply need to be immersed in zinc acetate solution; then, the acetate is volatilized after annealing and ZnO nanoparticles are formed inside the TiO_2_ NT micro-containers. The morphology and composition of the obtained ZnO@TiO_2_ heterojunction films were investigated by SEM (scanning electron microscopy), TEM (transmission electron microscopy), and XRD (X-ray diffraction). The photocatalytic performances of ZnO@TiO_2_ heterojunction films were also further evaluated by degradation of methyl orange solution.

## Experimental section

### 2.1 Fabrication of TiO_2_ NTs arrays

The titanium dioxide nanotubes were prepared by anodic oxidation reaction^38,39^ on pure titanium sheets with the size of 1.5 cm × 5 cm. The cut sheets were cleaned and soaked in ethanol for sonication. Electrolyte is required for the electrochemical reaction. As the reaction medium, the electrolyte must have corrosion property and the capability to provide oxygen ions. The electrolyte solution consists of 98% by volume of ethylene glycol and 2% by volume of deionized water with the addition of 0.3% by mass of ammonium fluoride. Two titanium sheets were selected as anode and cathode, which were connected to the positive and negative electrodes of the power supply, respectively. At the same time, the two electrodes separated by 2.5 cm were immersed in the electrolyte solution. The constant voltage between the two titanium sheets was 55 V and the reaction lasted for 2 h. Magnetic stirring was maintained during the reaction. TiO_2_ NTs arrays grew on the anode titanium sheet. After the reaction, TiO_2_ NTs arrays were rinsed and soaked in alcohol for 12 h. The nanotubes were dried in an oven at 80 °C. Then, it was annealed in a sintering furnace at a heating rate of 2 °C per minute and held for two hours after reaching 450 °C.

### 2.2 Fabrication of ZnO@TiO_2_ heterojunction films

**Fig. 1:**
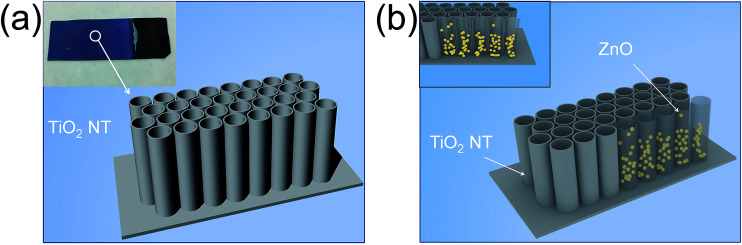
Structural diagram of anodic TiO_2_ nanotube arrays (a) and ZnO@TiO_2_ heterostructured film. (b) The empty TiO_2_ nanotubes were taken as nano-containers and nano-reactors for the formation of ZnO nanoparticles inside the TiO_2_ nanotubes.

TiO_2_ NTs arrays with metal oxide modification can achieve better performance. ZnO was selected as the metal oxide for modification in this study. The synthesis method is simple and efficient, which mainly involves a thermal decomposition process, which is schematically shown in Fig. 1. Zinc acetate was dissolved in deionized water at a concentration gradient of 0.2 mol L^−1^, 0.4 mol L^−1^, 0.6 mol L^−1^, 0.8 mol L^−1^, and 1.0 mol L^−1^. The prepared TiO_2_ NTs arrays were immersed in different concentrations of zinc acetate solution, while a large number of bubbles appeared on the film, which indicated that the solution entered the tube. After the bubbles disappeared, the sheets were taken out. The soaked TiO_2_ NTs arrays were gently wiped with a filter paper to remove the excess solution (if the excess solution on the titanium sheet is not removed, zinc oxide may cover the surface of the NTs arrays and thus affect their photocatalytic performance). The subsequent step is annealing, at a temperature of 400 °C for 2 h, followed by natural cooling. As shown in Fig. 1, the TiO_2_ NTs were first used as containers to load the zinc acetate solution; then, they acted as nanoreactors for the thermal decomposition of zinc acetate. In the end, ZnO nanoparticles were formed inside those TiO_2_ NTs and finally, ZnO@TiO_2_ heterojunction films were obtained. Other experimental details are shown in ESI.† Different concentrations of zinc acetate solution were used during the fabrication process and thus, various ZnO@TiO_2_ heterojunction films were obtained. For the simplicity of presentation, we denoted the ZnO@TiO_2_ heterojunction films as ZnO-0.2 (0.4, 0.6, 0.8, and 1), which corresponds to the TiO_2_ NTs arrays film impregnated with 0.2 (0.4, 0.6, 0.8, and 1) mol L^−1^ zinc acetate solution.

### 2.3 Photocatalytic performances of ZnO@TiO_2_ heterojunction films

The photocatalytic activity of ZnO@TiO_2_ heterojunction films were characterized by photodegradation of organic dyes in water. Methyl orange at a concentration of 10^−4^ mol L^−1^ was used the indicator for testing, which acts as an organic pollutant. The ZnO@TiO_2_ heterojunction films (cut to fixed size: 1.5 cm × 3 cm) were placed in a quartz cup filled with methyl orange solution (5 mL). They were magnetically stirred in the dark for 1 h to attain adsorption–desorption equilibrium. Then, they were irradiated by UV light with a power of 28 W and a light intensity of 10.5 mW cm^−2^. The methyl orange solution was sampled every half an hour. The above photodegradation tests were carried out on the heterojunctions separately. The concentration of the sampled methyl orange solution was measured using a spectrophotometer.

### 2.4 Characterization

For the observation of TiO_2_ NTs morphology and structure, a scanning electron microscope (SEM, JSM-7000F, JEOL Inc. Japan) was used. For the determination of crystallization degree of TiO_2_ and the ZnO crystallization in heterojunctions, X-ray diffraction (XRD) technique was used. For the comparison between TiO_2_ NTs arrays' and ZnO@TiO_2_ heterojunction films' ability to degrade organic pollutants under ultraviolet light, a spectrophotometer (JASCO V-570 UV/VIS/NIR) was used.

## Results and discussion

**Fig. 2:**
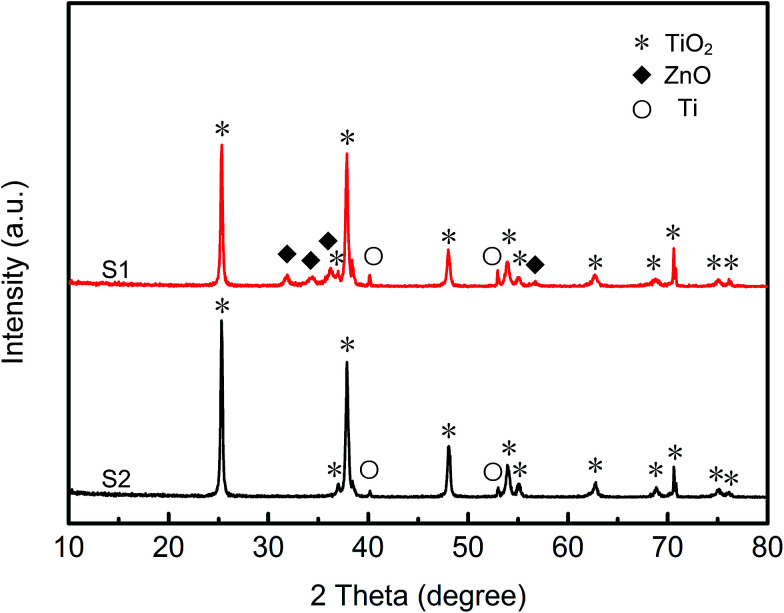
XRD pattern of TiO_2_ NTs arrays and ZnO@TiO_2_ heterojunctions. S1 stands for ZnO@TiO_2_ heterojunctions and S2 stands for TiO_2_ NTs arrays.

TiO_2_ NTs generally do not exhibit effective photocatalytic activity if they are amorphous. Hence, in this study, the TiO_2_ NTs arrays film produced by anodic oxidation were annealed for crystallization before loading ZnO crystals. X-ray diffraction patterns of the pure TiO_2_ NTs arrays and the ZnO@TiO_2_ heterojunction films are shown in Fig. 2. It can be seen that the pure TiO_2_ NTs arrays were well crystallized. The diffraction peaks at 25.2°, 36.9°, 37.8°, 48.1°, 53.89°, 55.0°, 62.68°, 75.0°, and 76.0° were attributed to the (101), (103), (004), (200), (105), (211), (204) (215), and (301) planes of anatase phase, respectively. Moreover, the minor diffraction peaks at 40.1° and 53.0° were attributed to (101) and (102) planes of Ti, respectively. These results indicate that the TiO_2_ NTs arrays were crystallized into anatase phase before they were used as containers or reactors to load ZnO. Compared with pure TiO_2_, the diffraction peaks of ZnO were observed in ZnO@TiO_2_ heterojunction films. New peaks at 31.7°, 34.4°, and 36.2° could be assigned to the (100), (002) and (101) planes of hexagonal ZnO, respectively.^40^ The lattice parameters of the ZnO can be obtained by calculation. As a hexagonal structure, the lattice parameters of ZnO are calculated as: *a* = *b* = 0.3253 nm, *c* = 0.5129 nm which are close to the reported values: *a* = *b* = 0.3249 nm and *c* = 0.5206 nm.^41^ It can be concluded from the XRD results that zinc acetate was thermally decomposed inside the TiO_2_ NTs arrays and hence, the ZnO@TiO_2_ heterojunction films were successfully obtained. XRD patterns of various ZnO@TiO_2_ heterojunctions are shown in ESI Fig. S2.† When zinc acetate solution at low concentration was used, the ZnO particles in the nanotubes were low in number and the diffraction peaks were not easily observed.

**Fig. 3:**
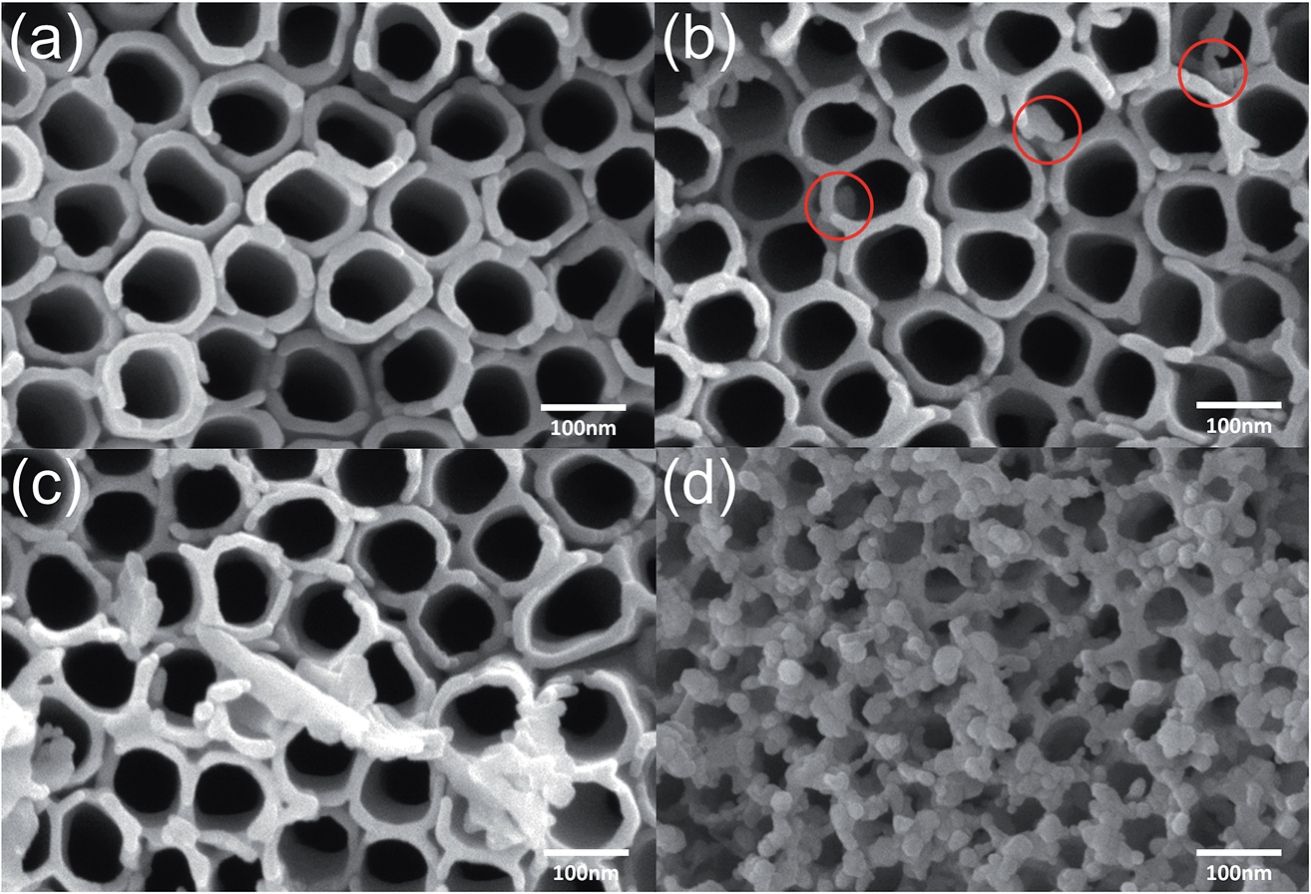
SEM images of TiO_2_ NTs and ZnO@TiO_2_ heterojunctions: (a) TiO_2_ NTs arrays. (b–d) SEM of heterojunctions with zinc acetate at various concentrations: 0.2 mol L^−1^, 0.6 mol L^−1^ and 1 mol L^−1^, respectively.

Microscopic morphology of the TiO_2_ NTs can be well observed by SEM. The microstructure of pure TiO_2_ nanotubes and nanotubes modified with ZnO are shown in Fig. 3(a). The as-prepared pure TiO_2_ NTs arrays are closely ordered with minute voids between the tubes; the nozzles are spherical in shape and the diameter of the tubes is approximately the same with an average of about 80 nm. The tube wall is about 10 nm thick. The morphology of the TiO_2_ NTs arrays film impregnated with low concentration zinc acetate solution has hardly been affected. At a concentration of 0.2 mol L^−1^ zinc acetate solution, as shown in Fig. 3(b), the red circles indicate the ZnO decorative particles, which are coated along the tube wall at the part of the nozzle. The open tube-mouth morphology is not affected. With the increase in concentration of zinc acetate, the nozzles of the TiO_2_ NTs are occupied by ZnO blocks and the nozzle area became smaller. When the concentration of zinc acetate solution was 0.6 mol L^−1^, it can be observed from Fig. 3(c) that most of the nanotubes were wide open, some nanotubes were covered, and the ZnO particles aggregated to form large blocks deposited around the nozzles. Fig. 3(d) shows that the tubular structure is almost invisible and replaced by the mesh structure and attached ZnO particles when the concentration of zinc acetate was 1 mol L^−1^. The above SEM results indicate that the ZnO@TiO_2_ heterojunctions can be prepared by a simple thermal decomposition method and the ZnO particles were directly observed through SEM. The loading amount of ZnO is directly related to the increase in the concentration of zinc acetate. With an appropriate amount of ZnO particles, the morphology of TiO_2_ nanotubes will not be affected. However, on continuously increasing the loading amount of ZnO, the nozzle was gradually filled until it was completely covered and the open-tube mouth morphology and tubular structure disappeared.

**Fig. 4:**
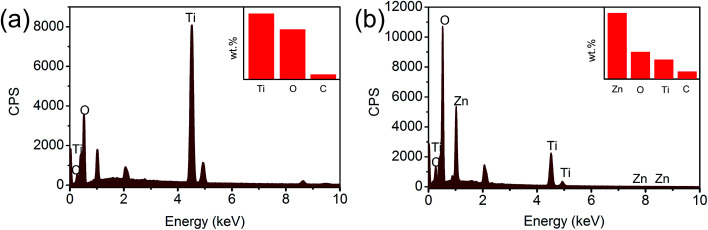
(a) EDS diagram of pure TiO_2_ NTs arrays and (b) the ZnO-0.8 heterojunction films.

In order to further confirm the preparation of the ZnO@TiO_2_ heterojunction films, energy-dispersive spectrometer (EDS) was adopted for elemental analysis. Fig. 4(a) shows the EDS results of pure TiO_2_ NTs arrays. The characteristic peaks of only Ti, O and a small amount of C could be observed, indicating the formation of anatase TiO_2_ NTs. Fig. 4(b) shows the EDS results of the ZnO-0.8 heterojunction films, in which the characteristic peak of Zn is clearly observed. The inset of Fig. 4(b) is the calculated elements proportion, in which Zn is shown as the main element of the film since EDS is a surface analytic technology. More elemental descriptions are listed in ESI Tables S1 and S2.† The main elements in TiO_2_ nanotubes were Ti and O, whose atom percentages were 27.94% and 65.17%, respectively. In ZnO@TiO_2_ heterojunction, the atomic percentage of Zn reached 27% and the mass percentage reached 55%. Nevertheless, the EDS results confirm that ZnO were successfully loaded in the TiO_2_ NTs arrays.

**Fig. 5:**
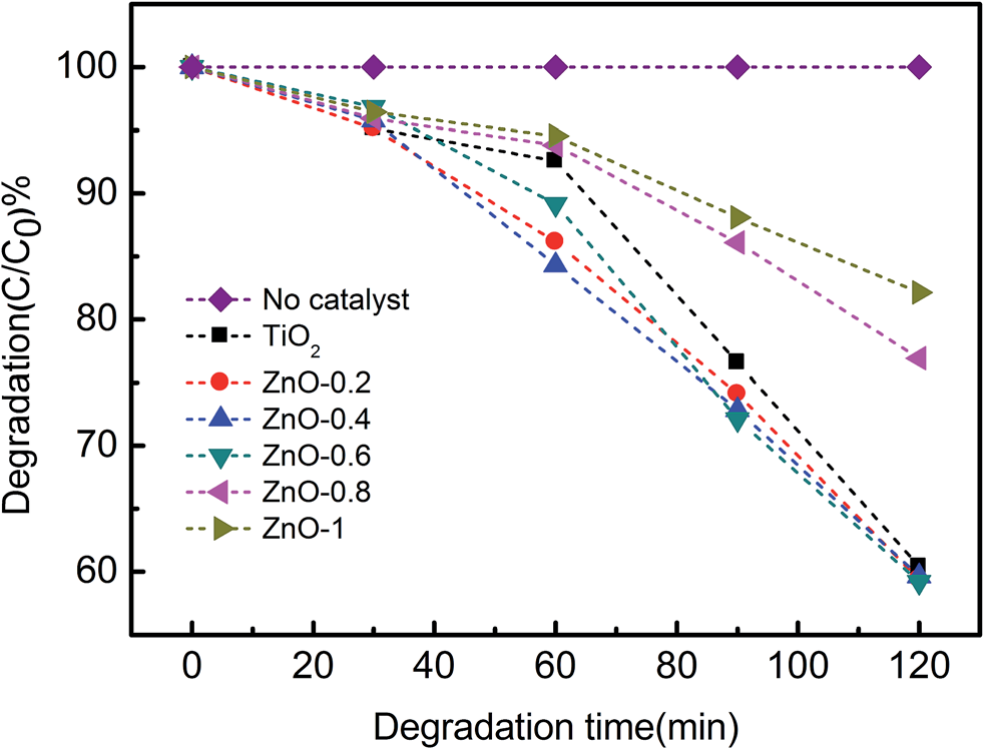
Degradation kinetics of methyl orange solution by TiO_2_ NTs films and various types of ZnO@TiO_2_ heterojunction films.

The photocatalytic properties of the as-prepared ZnO@TiO_2_ heterojunction films were evaluated by degradation of methyl orange solution. The methyl orange solution acts as the probe organic pollutant with an initial concentration of 10^−4^ mol L^−1^ (*C*_0_). The heterojunction films were immersed into the methyl orange solution for photodegradation. Then, the methyl orange solution was sampled every 30 min. The concentration of methyl orange (labeled as *C*) was measured with the increase in degradation time. The decomposition degree of methyl orange can be expressed as *C*/*C*_0_. The environment for photodegradation of methyl orange is shown in ESI Fig. S1.† As shown in Fig. 5, methyl orange could also be subjected to photocatalytic degradation by the pure TiO_2_ NTs arrays since the TiO_2_ NTs arrays are essentially photocatalysts. For the ZnO@TiO_2_ heterojunction films, samples ZnO-0.2, ZnO-0.4, and ZnO-0.6 exhibited enhanced photocatalytic performances. In the first 60 min, the enhanced photocatalytic activities were in the order of sample ZnO-0.4, sample ZnO-0.2, and sample ZnO-0.6. After 90 min, sample ZnO-0.6 exhibited the best performance. Finally, after 120 min, all the above three samples had shown improved photocatalytic activities as compared to pure TiO_2_ NTs arrays. Moreover, the degradation kinetics, as shown in Fig. 5, indicated that samples ZnO-0.8 and ZnO-1 had deteriorated photocatalytic activities as compared to pure TiO_2_ NTs arrays.

**Fig. 6:**
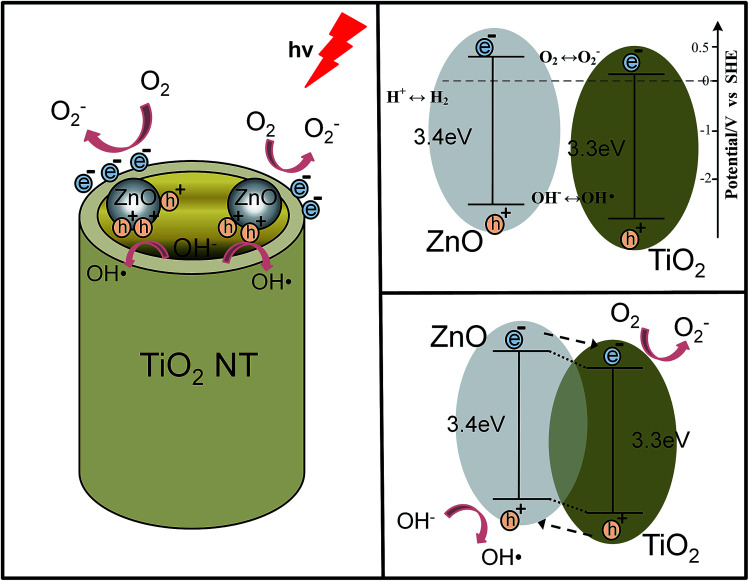
Mechanism of photocatalysis. (a) Electron transfer to the material surface and the generation of reactive oxygen species. (b) The relative positions of Fermi energy before ZnO and TiO_2_ contact. (c) Electron hole separation in heterojunctions.

The above photocatalytic results indicate that the ZnO@TiO_2_ heterojunction films have improved degradation performances than that of the pure TiO_2_ NTs arrays when an appropriate amount of ZnO nanoparticles were loaded (or an appropriate concentration of zinc acetate solution was used). The mechanism of the ZnO@TiO_2_ heterojunction films with enhanced photocatalytic degradation is demonstrated in Fig. 6. In particular, titanium dioxide (TiO_2_), a wide-band-gap semiconductor (∼3.2 eV), is useful for organic degradation. When TiO_2_ was appropriately irradiated, electron–hole pairs or excitons could be generated by incoming photons, which either migrate to the material surface or recombine and dissipate the energy as heat. Those on the surface can then participate in redox reactions (react with O_2_ to form O_2_^−^) and generate reactive oxygen species (ROS),^42,43^ while holes oxidize OH^−^ to yield hydroxyl radicals (OH˙).^44^ These radicals play an important role in reacting with the methyl orange molecules and decompose them into CO_2_ and H_2_O. Before ZnO and TiO_2_ contact, the relative positions of their conduction bands and valence bands are shown in Fig. 6(b). ZnO and TiO_2_ have a similar bandgap value and only ultraviolet radiation can excite the electrons. When ZnO was loaded to the TiO_2_ NTs arrays and formed ZnO@TiO_2_ heterojunctions, photogenerated electron–hole separation occurs on both TiO_2_ and ZnO separately under ultraviolet light. The heterojunction between TiO_2_ and ZnO led to the crossing of their energy levels. The photogenerated electrons transferred from the ZnO conduction band to the TiO_2_ conduction band and the holes moved from TiO_2_ valence band to ZnO valence band as shown in Fig. 6(c). Photogenerated electrons will be separated in this case, while the recombination will be suppressed,^45^ resulting in accelerating the formation of ROS as shown in Fig. 6(a). These highly reactive ROS reacted with the methyl orange molecules and decomposed them into CO_2_ and H_2_O. Photocatalytic testing indicated that with an appropriate zinc acetate concentration (ZnO-0.2, ZnO-0.4, and ZnO-0.6), the role of ZnO modification is positive for the ZnO@TiO_2_ heterojunction films. SEM results have shown that the ZnO is attached only to the tube wall without clogging the nozzle. Compared with pure TiO_2_ NTs, the deteriorated activities of samples ZnO-0.8 and ZnO-1 were attributed to the excessive loaded ZnO nanoparticles. SEM results have also clearly shown that the overdose of ZnO crystals block the entire tube-mouth and surface of the TiO_2_ NTs arrays (see Fig. 3(d)). Without open-tube mouths, both the MO molecules transportation and photo-generated ROS diffusion will be restricted. Therefore, there should be a balance between ZnO loading mass and retaining the open tube-mouth configuration for achieving the best photocatalytic activity of the ZnO@TiO_2_ heterojunction films. Since the above discussion is consistent with XRD, SEM, EDS, and photocatalytic results, we believe that the optimized ZnO@TiO_2_ heterojunction films exhibited enhanced photocatalytic activities as compared to pure TiO_2_ NTs arrays.

## Conclusions

Compact and ordered TiO_2_ NTs arrays were used as substrates and modified with a certain amount of ZnO crystalline particles. After a simple thermal decomposition process, a series of ZnO@TiO_2_ heterojunctions films were successfully prepared. The heterojunction obtained by this method is simple and efficient. The zinc acetate was transferred to the inner walls and periphery of the nanotubes when the TiO_2_ NTs arrays were used as nano-containers and nano-reactors. The morphological and crystal properties of the final ZnO@TiO_2_ heterojunctions films could be tuned by using different concentrations of zinc acetate. SEM results indicated that an excessive concentration of zinc acetate blocked the TiO_2_ NT mouth morphology, destroyed the tubular structures, and deteriorated the photocatalytic activities. The heterojunction obtained using zinc acetate with concentrations of 0.4 and 0.6 mol L^−1^ had a much improved photocatalytic effect as compared to TiO_2_ NTs arrays, which could be attributed to the separation of electron–hole pairs and the inhibition of recombination. The ZnO@TiO_2_ heterojunctions films prepared in this study are expected to be used as efficient recyclable photocatalysts or used in other environmental and energy related areas.

## Conflicts of interest

There are no conflicts to declare.

## Supplementary Material

RA-008-C7RA13222K-s001
