# Supplementary material for: Preparation of ZnO@TiO2 nanotubes heterostructured film by thermal decomposition and their photocatalytic performances
Source: RSC Adv. 2018 Feb 20;8(15):8064–70. doi: 10.1039/c7ra13222k (PMC9078507; doi:10.1039/c7ra13222k)
Supplement: RA-008-C7RA13222K-s001 [file RA-008-C7RA13222K-s001.pdf]

Electronic Supplementary Material (ESI) for RSC Advances.
This journal is © The Royal Society of Chemistry 2018

Supporting Information

Preparation of $ZnO@TiO_2$ nanotubes heterostructured film by thermal decomposition and their photocatalytic performances

Yulong Liao, [a, b 1] *Kaibin Zhang,* [a] *Dainan Zhang,* [a] *Yuanxun Li,* [a] *Hua Su,* [a] *Huaiwu Zhang,* [a] *and Zhiyong Zhong* [a]

[a] State Key Laboratory of Electronic Thin Film and Integrated Devices, University of Electronic Science and Technology of China, Chengdu 610054, China.

[b] Center for Applied Chemistry, University of Electronic Science and Technology of China, Chengdu 610054, China.

[*] Corresponding author:

Tel.: +86-028-83201440; Fax: +86-028-83202556

Email address: yulong.liao@uestc.edu.cn

To whom correspondence should be addressed, E-mail: yulong.liao@gmail.com, hwzhang@uestc.edu.cn.

Experimental Details:

Preparation:The required size of titanium sheet for anodic oxidation was 1.5 cm×5 cm. After cutting, the sheets were flattened by pressing. Titanium sheets were immersed in electrolyte solution 4cm, the relative distance was 2.5cm. The electrolyte consists of 98% volume ratio of ethylene glycol, deionized water of 2% volume ratio and 0.3% mass ratio of ammonia fluoride. The constant voltage between the two titanium plates is 55v, and the reaction lasted 2 hours. The temperature of the reaction was controlled to be 30 ^{o}C in the reaction process. High or low temperature would hinder the growth of nanotubes and make them fall off. TiO_2 NTs arrays obtained by anodic oxidation were annealed for crystallization. The annealing temperature is 450 ^{o}C for 2 hours and natural cooling Zinc acetate was dissolved in deionized water at a concentration gradient of 0.2 mol/L, 0.4 mol/L, 0.6 mol/L, 0.8 mol/L, 1 mol/L. Various samples were obtained by immersing the nanotubes in different concentrations of zinc acetate solution. The samples were identified: ZnO-0.2, ZnO-0.4, ZnO-0.6 ZnO-0.8 ZnO-1. The soaked films were taken out and the exceeded solution on the titanium sheet was removed with filter paper to prevent the ZnO from covering the surface of the NTs arrays. The subsequent step is annealing, the annealing temperature is 400 ^{o}C for 2 hours and natural cooling.

Characterization: For the observation of TiO_2 NTs morphology and structure, a scanning electron Microscopy (SEM, JSM-7000F, JEOL Inc. Japan) is used. For the crystallization degree of TiO_2 and the determination of ZnO crystallization in heterojunctions, X-ray diffraction (XRD) is used. For the

comparison between TiO_2 and heterojunction of the ability to degrade organic pollutants under ultraviolet light, a spectrophotometer (JASCO V-570 UV/VIS/NIR) is used

Photocatalytic testing: The environment for photodegradation of methyl orange is shown in Fig S1.In this experiment, methyl orange solution was used as organic pollutant, and the initial concentration was 10^{-4}mol/l. Methyl orange solution (5mL) was placed into a quartz cup. The area of the $ZnO@TiO_2$ heterojunction films immersed in methyl orange solution was 1.5 cm×3 cm. They were magnetically stirred in dark for 1 hour to reach the adsorption-desorption equilibrium. The UV lamp with rated power of 28w was used to irradiate the solution, and the methyl orange solution was sampled every half hour for four times. The concentration of methyl orange solution was measured with spectrophotometer, and the photodegradation ability of the film could be expressed as the initial concentration divided by the concentration after degradation (C/C_0).

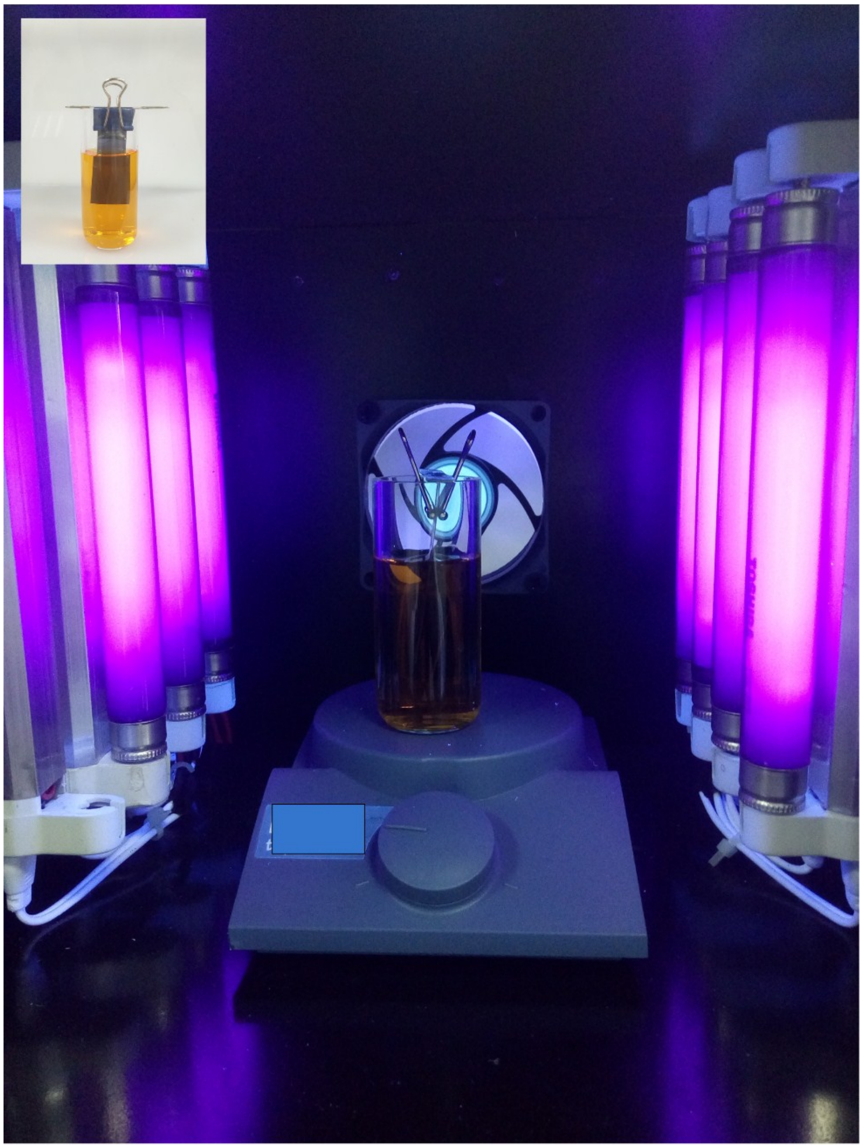

Figure S1. Equipment for photodegradation.

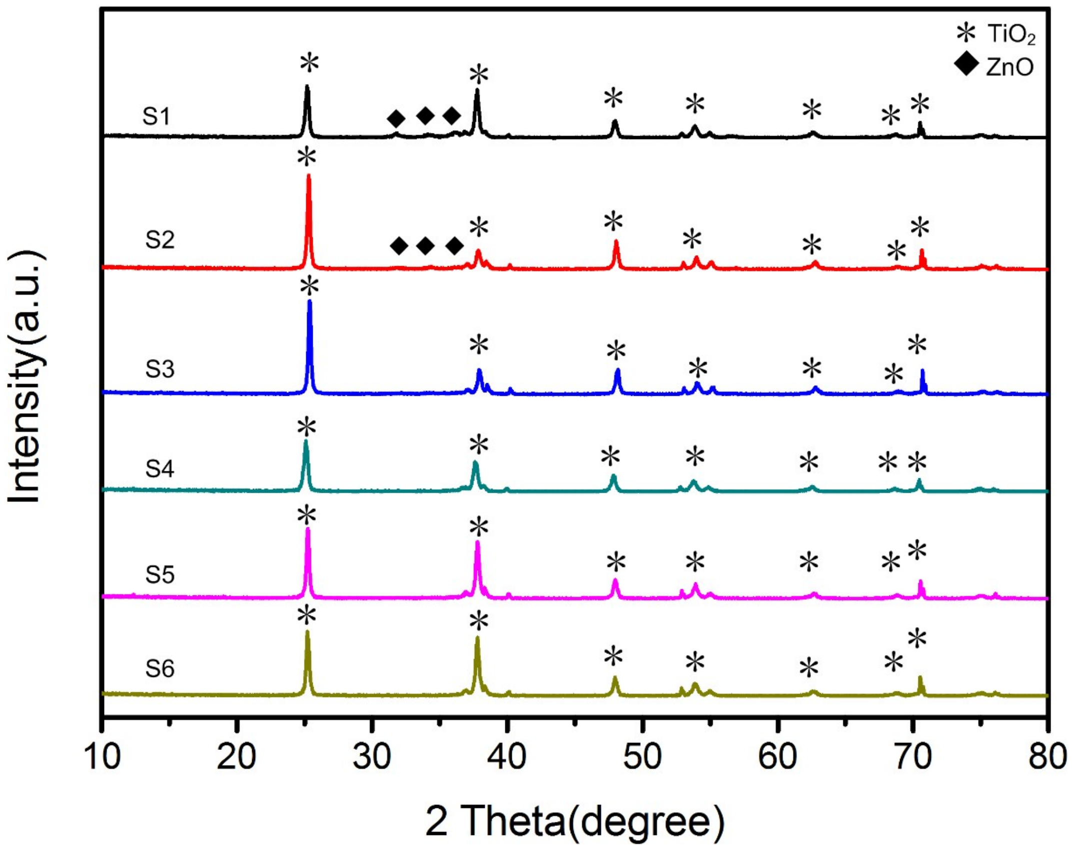

Figure S2. XRD patterns of partial TiO_2&ZnO heterojunction films. S1,S2,S3,S4,S5,S6 stand for ZnO-1.2, ZnO-1, ZnO-0.8, ZnO-0.6, ZnO-0.4, ZnO-0.2 films.

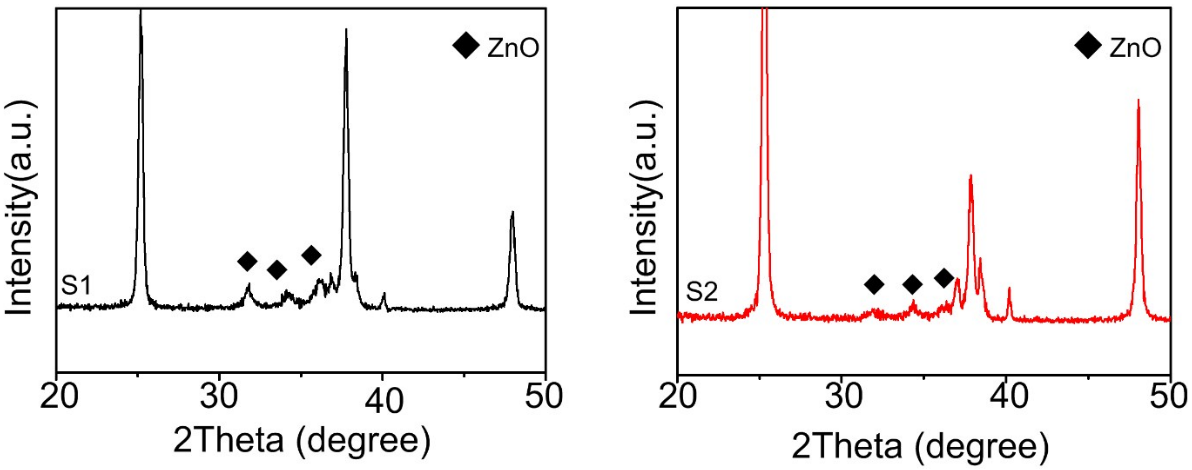

Figure S3. ZnO diffraction peak enlargement of ZnO-1.2(S1), ZnO-1(S2) films.

Table S1. Element Contents Based on EDS Results of TiO_2 NTs

element	Apparent concentration	K ratio	wt%	wt% Sigma	Atomic percentage	Standard sample label
C	0.73	0.00730	3.36	0.19	6.90	C Vit
O	11.94	0.04018	42.32	0.37	65.17	SiO_2
Ti	42.24	0.42242	54.32	0.36	27.94	Ti

Table S2. Element Contents Based on EDS Results of $ZnO@TiO_2$ heterojunctions

element	Apparent concentration	K ratio	wt%	wt% Sigma	Atomic percentage	Standard sample label
C	0.29	0.00292	5.26	2.08	14.36	C Vit
O	6.67	0.02243	22.64	8.93	46.40	SiO_2
Ti	4.53	0.04535	16.76	6.61	11.48	Ti
Zn	14.27	0.14267	55.34	17.61	27.76	Zn
